# Real-world effectiveness of guselkumab for induction of remission in Crohn’s disease: a prospective multicenter cohort study with propensity score–matched comparison to risankizumab

**DOI:** 10.1093/crocol/otag061

**Published:** 2026-06-19

**Authors:** Maitha Al Hosani, Noorah Al Hosani, Mohamed Nasir Alzaabi, Maryam A Alahmad, Nadeen Mamon Omar, Camille Conde Oribiana, Enas Fouad Ahmed, Ishtiaq Ahmed, Kishore Kumar Chitra Kumar, Sofia Hanif, Khalid Izzeldin Elfatih Barakat, Khalid Osman Elamin Elsayed, Mohammed Nabil Quraishi

**Affiliations:** Department of Gastroenterology, Sheikh Shakhbout Medical City, Abu Dhabi, United Arab Emirates; Department of Gastroenterology, Sheikh Shakhbout Medical City, Abu Dhabi, United Arab Emirates; Department of Gastroenterology, Sheikh Shakhbout Medical City, Abu Dhabi, United Arab Emirates; Department of Gastroenterology, Sheikh Shakhbout Medical City, Abu Dhabi, United Arab Emirates; Department of Gastroenterology, Sheikh Shakhbout Medical City, Abu Dhabi, United Arab Emirates; Department of Gastroenterology, Sheikh Shakhbout Medical City, Abu Dhabi, United Arab Emirates; Department of Gastroenterology, Sheikh Shakhbout Medical City, Abu Dhabi, United Arab Emirates; Department of Gastroenterology, Sheikh Shakhbout Medical City, Abu Dhabi, United Arab Emirates; Department of Gastroenterology, Sheikh Shakhbout Medical City, Abu Dhabi, United Arab Emirates; Burjeel Institute of Digestive Health, Burjeel Hospital, Abu Dhabi, United Arab Emirates; Burjeel Institute of Digestive Health, Burjeel Hospital, Abu Dhabi, United Arab Emirates; Burjeel Institute of Digestive Health, Burjeel Hospital, Abu Dhabi, United Arab Emirates; Department of Gastroenterology, Sheikh Shakhbout Medical City, Abu Dhabi, United Arab Emirates; Institute of Cancer and Genomic Sciences, University of Birmingham, Birmingham, United Kingdom; College of Medicine and Health Sciences, Khalifa University of Science and Technology, Abu Dhabi, United Arab Emirates

**Keywords:** guselkumab, risankizumab, Crohn’s disease, inflammatory bowel disease, real-world evidence, comparative effectiveness, propensity score matching, IL-23 inhibitors

## Abstract

**Background:**

No head-to-head trials compare selective interleukin-23 inhibitors in Crohn’s disease (CD), leaving clinicians without direct evidence to guide treatment selection. We aimed to evaluate the real-world induction effectiveness of guselkumab in CD and perform propensity score–matched (PSM) comparisons against risankizumab.

**Methods:**

This prospective multicenter study included adults with moderate-to-severe CD initiating guselkumab in the United Arab Emirates. The primary outcome was clinical remission at Week 12 (Harvey-Bradshaw Index <5). Full propensity score matching between guselkumab and risankizumab cohorts controlled for 12 covariates, including disease phenotype, baseline endoscopic and biochemical activity, surgical history, and current smoking status.

**Results:**

Fifty-one guselkumab and 60 risankizumab patients were included. In the guselkumab cohort, 71% were advanced therapy-exposed with a median of 2 prior advanced therapies. At Week 12, clinical remission was achieved in 51% (26/51) and clinical response in 86% (44/51). Median C-reactive protein (CRP) decreased from 8.5 to 3.0 mg/L (*P* = .002) and fecal calprotectin from 216 to 80 μg/g (*P* < .001). Effectiveness was consistent regardless of prior treatment history. On univariable analysis, higher baseline HBI (OR 0.77, *P* = .011), SES-CD (OR 0.87, *P* = .049), and CRP (OR 0.94, *P* = .019) were associated with lower remission likelihood. Maintenance dosing was 100 mg Q8W in 73% and 200 mg Q4W in 27%. PSM analysis demonstrated comparable clinical remission (51% vs 50%, *P* > 0.9) and clinical response (86% vs 87%, *P* = .87) between guselkumab and risankizumab, with no significant between-group biomarker differences.

**Conclusions:**

Guselkumab demonstrated robust real-world induction effectiveness comparable to risankizumab across clinical and biochemical outcomes. These findings warrant confirmation in larger, longer-term comparative studies with endoscopic endpoints.

## Introduction

The therapeutic landscape for inflammatory bowel disease (IBD) has been transformed by selective interleukin-23 (IL-23) p19 inhibitors, which represent a mechanistically refined approach to targeting chronic intestinal inflammation.^1^^,^^2^ The central role of IL-23 in IBD pathogenesis has been validated by the clinical success of agents blocking this pathway.^3^^,^^4^ Unlike ustekinumab, which targets the shared p40 subunit of IL-12 and IL-23, selective p19 inhibitors—guselkumab, risankizumab, and mirikizumab preserve IL-12 signaling critical for host defence.^5^^,^^6^ This selective blockade has demonstrated clinical advantages, with the SEQUENCE trial suggesting superiority of risankizumab over ustekinumab for endoscopic outcomes in anti-TNF-exposed Crohn’s disease (CD), and the GALAXI trials demonstrating similar benefits for guselkumab.^7^^,^^8^

Despite shared targets, these agents possess distinct molecular and pharmacological profiles that may translate into clinical differences.^9^^,^^10^ Recent network meta-analyses have positioned guselkumab and risankizumab favorably within the CD therapeutic hierarchy, though whether these molecular differences translate into differential real-world effectiveness remains under investigation.^11^^,^^12^ A critical evidence gap persists: no randomized controlled trials have directly compared IL-23 p19 inhibitors against one another. The head-to-head IBD biologic trials conducted to date have not addressed intra-class comparisons among selective IL-23 inhibitors.^7^^,^^8^^,^^13–15^ Network meta-analyses are constrained by heterogeneity in trial designs, patient populations, and endpoint definitions, coupled with transitivity violations, leaving clinicians without direct comparative data to guide treatment positioning.^16^ Real-world evidence using propensity score methods has emerged as a rigorous approach to generate comparative effectiveness data from observational cohorts when randomized trials are unavailable.^17^^,^^18^

The United Arab Emirates (UAE) offers a unique environment for addressing this gap, with regulatory approval for guselkumab granted soon after regulatory approval in the United States, enabling on-label use for CD and facilitating rapid clinical adoption since April 2025. Within the UAE healthcare environment, Emirati nationals (who comprise the majority of our IBD population at SSMC) receive comprehensive national health coverage that supports access to advanced therapies, including guselkumab and risankizumab, without payer-mandated sequencing of selective IL-23 inhibitors. IBD in the Middle East exhibits a distinct phenotype characterized by aggressive disease behavior, earlier age of onset, and high rates of perianal and penetrating complications; features that predict poorer treatment outcomes and are underrepresented in pivotal trials.^19^^,^^20^ We have previously reported on the real-world effectiveness of risankizumab in this population, demonstrating robust post-induction and maintenance clinical effectiveness outcomes.^21^ The emergence of guselkumab as an additional IL-23 inhibitor creates an opportunity for a direct comparative effectiveness analysis between these agents.

In this study, we evaluate the real-world effectiveness of guselkumab induction in a multicenter cohort of patients with CD and perform a propensity score–matched comparative effectiveness analysis of guselkumab versus risankizumab.

## Methods

### Study design and population

This prospective, multicenter, observational cohort study utilized data from prospectively maintained IBD registries at two hospitals in Abu Dhabi, UAE. The study was approved by the Institutional Review Board (IRB) of both institutions (SSMCREC-585; date approved 2nd March 2025). All data used in this analysis were anonymized to protect patient confidentiality. Informed consent was waived by both IRBs as data were collected as part of a prospective clinical registry evaluating standard-of-care treatments. The study was conducted in accordance with the Declaration of Helsinki and reported following STROBE guidelines.^22^

Patients were eligible if they had moderately-to-severely active Crohn’s disease at the time of guselkumab initiation, defined as clinical activity (Harvey-Bradshaw Index ≥5) supported by at least one objective marker of inflammation, ascertained from one or more of: elevated C-reactive protein (>5 mg/L), elevated fecal calprotectin (>250 µg/g), and/or active inflammation on the most recent ileocolonoscopy (SES-CD ≥4) or magnetic resonance enterography prior to initiation. Consecutive adults (18 years or older) initiating guselkumab between April 2025 and October 2025 were eligible for inclusion; patients who received off-label guselkumab prior to regulatory approval in the UAE were also included. Patients with isolated perianal Crohn’s disease were excluded to allow consistent measurement and comparison of luminal disease activity outcomes; patients with perianal disease were therefore eligible only if they had concurrent luminal involvement. Patients switched from a prior advanced therapy due to intolerance and patients commenced for postoperative recurrence were similarly required to have objective evidence of active luminal disease at the time of guselkumab initiation as documented in the medical record. Inclusion was therefore not based on elevated HBI alone in any patient. Patients were required to have a minimum of 12 weeks of follow-up for post-induction assessment.

The comparator risankizumab cohort comprised 60 CD patients from our previously published single-center study.^21^ Baseline characteristics and outcomes for this cohort have been reported in full elsewhere. For the present analysis, individual patient-level data from the original dataset were used for propensity score matching.

### Treatment regimens

Guselkumab induction was administered as either intravenous 200 mg or subcutaneous 400 mg at weeks 0, 4, and 8, in accordance with the approved dosing schedule. The choice of post-induction maintenance regimen (100 mg subcutaneously every 8 weeks or intensified 200 mg subcutaneously every 4 weeks) was made at the Week 12 induction assessment based on response, and is reported in the Results as a marker of induction response. The first induction dose (Week 0) was administered in a supervised setting for all patients and documented in the electronic medical record. Subsequent doses at Weeks 4 and 8 were administered in the specialist IBD nurse-led clinic, by a home-care nursing team, or self-administered after training; pharmacy refill records were used to verify compliance, with subsequent doses dispensed only after documentation of the preceding administration. Concomitant oral corticosteroid use at baseline and during induction was at the discretion of the treating clinician, in keeping with real-world practice.

The risankizumab cohort received the standard induction regimen of 600 mg IV at weeks 0, 4, and 8, followed by maintenance at 360 mg SC Q8W, as previously described.^21^

### Outcomes

The primary outcome was clinical remission at Week 12, defined as Harvey-Bradshaw Index (HBI) <5. Secondary outcomes included clinical response (HBI reduction of 3 or more points from baseline), biochemical remission (C-reactive protein [CRP] <5 mg/L and fecal calprotectin [FCP] <250 μg/g), and changes in CRP and FCP from baseline to Week 12. Predictors of clinical remission were assessed using univariable logistic regression. Predictors of intensified guselkumab maintenance dosing selection were assessed separately.

### Statistical analysis

All analyses were conducted in R (version 4.4.2, R Foundation for Statistical Computing, Vienna, Austria) using RStudio (version 2024.12.1).^23^ Continuous variables were summarized as medians with interquartile ranges (IQR), and categorical variables as frequencies with percentages. Comparisons between advanced therapy (AT)-naive and AT-exposed groups were performed using the Wilcoxon rank-sum test for continuous data and Chi-squared or Fisher’s exact tests for categorical data as appropriate. Changes in inflammatory biomarkers from baseline to Week 12 were analyzed using the Wilcoxon signed-rank test for paired data. Statistical significance for all analyses was defined as *P* of .05 or less. The comparative analysis between guselkumab and risankizumab cohorts was descriptive and hypothesis-generating; no a priori sample size calculation was performed. The guselkumab cohort comprised all consecutive eligible patients during the prospective registry window, and the risankizumab comparator was the entirety of our previously published single-center cohort.

Univariable logistic regression was used to identify predictors of induction outcomes. Variables with *P* < 0.1 on univariable analysis were entered into a multivariable logistic regression model. Variables were selected a priori based on clinical relevance in predicting treatment outcomes in CD. For clinical remission, the model incorporated age, gender, disease duration, baseline HBI, baseline SES-CD, baseline CRP, baseline fecal calprotectin, number of prior advanced therapies, perianal disease, prior CD surgery, AT-naive status, and individual prior biologic class exposures (anti-TNF, ustekinumab, vedolizumab, upadacitinib, and risankizumab).

### Propensity score matching

For the comparative analysis, CD patients treated with guselkumab were matched to CD patients treated with risankizumab from our previously published cohort.^21^ Propensity scores were estimated using logistic regression with 12 covariates: age, gender, disease duration, Montreal classification (location and behavior), perianal disease, total prior advanced therapy exposure, baseline CRP, baseline fecal calprotectin, baseline SES-CD, prior CD surgery, and current smoking status (current vs not-current). Full (optimal) matching was performed using the MatchIt package,^24^ which creates variable-ratio matched sets and retains all patients in both treatment arms, maximizing statistical power in a setting with modest sample sizes. Post-matching, each patient received a weight reflecting the structure of the matched sets. All subsequent comparisons used these weights, with weighted logistic regression for clinical outcomes and weighted linear regression for between-group biomarker comparisons. Within-group biomarker changes were assessed using the Wilcoxon signed-rank test for paired data.

For the primary PSM analysis, guselkumab-treated CD patients with prior risankizumab exposure were included if risankizumab had been discontinued due to intolerance, hypersensitivity, or unclear treatment response rather than confirmed treatment failure, as these patients could reasonably be expected to benefit from an alternative IL-23 p19 inhibitor. A pre-specified sensitivity analysis excluding all patients with prior risankizumab exposure (*n* = 7) was performed to account for potential carryover effects or within-class switching bias.

### Ethical considerations

This study was approved by the institutional ethics committees of both participating institutions (SSMCREC-585). Individual patient consent was waived, given the retrospective, observational design with use of routinely collected clinical data.

## Results

### Guselkumab cohort characteristics

Fifty-one patients with CD completed the Week 12 assessment and were included in the primary effectiveness analysis (Table 1). The cohort reflected a highly refractory population: 71% (36/51) had prior exposure to at least one advanced therapy, with a median of 2 prior advanced therapies (IQR 1-4). Prior exposures included anti-TNF agents in 59% (30/51), ustekinumab in 35% (18/51), risankizumab in 14% (7/51), vedolizumab in 14% (7/51), and upadacitinib in 12% (6/51). Seven patients (14%) had received prior risankizumab, all of whom had discontinued due to intolerance or unclear response rather than confirmed treatment failure.

**Table 1:** Guselkumab Crohn’s disease cohort baseline characteristics.

Characteristic	Overall (*N* = 51)	AT-naive (*N* = 15)	AT-exposed (*N* = 36)	*P*-value
**Age (years)**	32 (23, 37)	29 (23, 36)	36 (24, 37)	.62
**Disease duration (months)**	85 (26, 125)	16 (4, 110)	99 (46, 139)	.003
**Baseline HBI**	6.0 (5.0, 8.0)	6.0 (5.0, 7.0)	7.0 (4.0, 9.0)	.34
**Baseline CRP (mg/L)**	8 (3, 23)	8 (1, 23)	8 (4, 22)	.62
**Baseline albumin (g/L)**	41 (39, 44)	42 (39, 44)	41 (38.5, 44)	.69
**Baseline FCP (**µ**g/g)**	239 (80, 801)	684 (80, 1390)	202 (77, 726)	.24
**Baseline SES-CD**	6.0 (5.0, 9.0)	7.0 (5.0, 11.0)	6.0 (5.0, 8.5)	.23
**Baseline hemoglobin (g/L)**	135 (118, 141)	130 (114, 141)	136 (119, 143)	.68
**No. of prior ATs**	2 (1, 4)	1 (1, 1)	3 (2, 5)	<.001
**Male, *n* (%)**	33 (65)	9 (60)	24 (67)	.65
**Montreal L3, *n* (%)**	38 (75)	7 (47)	31 (86)	.012
**Montreal B1, *n* (%)**	32 (63)	14 (93)	18 (50)	.012
**Perianal disease, *n* (%)**	16 (31)	1 (7)	15 (42)	.019
**Prior CD Surgery, *n* (%)**	13 (25)	0 (0)	13 (36)	.006
**Prior anti-TNF, *n* (%)**	30 (59)	0 (0)	30 (83)	<.001
**Prior ustekinumab, *n* (%)**	18 (35)	0 (0)	18 (50)	<.001
**Prior vedolizumab, *n* (%)**	7 (14)	0 (0)	7 (19)	.090
**Prior upadacitinib, *n* (%)**	6 (12)	0 (0)	6 (17)	.16
**Prior risankizumab, *n* (%)**	7 (14)	0 (0)	7 (19)	.090
**Current smoker, *n* (%)**	5 (9.8)	1 (6.7)	4 (11)	>.99
**Ex-smoker, *n* (%)**	4 (7.8)	1 (6.7)	3 (8.3)	—
**Non-smoker, *n* (%)**	42 (82)	13 (87)	29 (81)	—

Continuous variables: median (IQR). Categorical variables: *n* (%). *P* value: Wilcoxon rank-sum, Pearson Chi-squared, or Fisher’s exact test. AT = advanced therapy; CRP = C-reactive protein; FCP = fecal calprotectin; HBI = Harvey-Bradshaw Index; SES-CD = simple endoscopic score for Crohn’s disease.

The median age was 32 years (IQR 23-37) with 65% male. The median disease duration prior to guselkumab initiation was 85 months (IQR 26-125), and the median age at diagnosis was 23 years (IQR 19-28). Ileo-colonic disease (Montreal L3) was predominant at 75% (38/51), with inflammatory behavior (Montreal B1) in 63% (32/51) and perianal disease in 31% (16/51). Prior CD surgery had been performed in 25% (13/51). Baseline disease activity was moderate, with a median HBI of 6.0 (IQR 5.0-8.0), CRP of 8 mg/L (IQR 3-23), albumin of 41 g/L (IQR 39-44), fecal calprotectin of 239 μg/g (IQR 80-801), and SES-CD of 6.0 (IQR 5.0-9.0). Concomitant oral corticosteroid use at baseline was minimal in both cohorts. Among the small number of guselkumab patients receiving baseline corticosteroids, the majority had completed tapering and were off corticosteroids by Week 12; one patient remained on a weaning steroid course at Week 12 in association with an inadequate induction response. No patients were receiving concomitant immunomodulators. Five (10%) of patients on guselkumab were current smokers at baseline, compared with 14 (23%) of patients on risankizumab; smoking status was included as a covariate in the propensity score–matched analysis.

Comparison between AT-naive (*n* = 15, 29%) and AT-exposed (*n* = 36, 71%) subgroups revealed that AT-naive patients had significantly shorter disease duration (median 16 vs 99 months, *P* = .003), fewer prior advanced therapies (median 1 vs 3, *P* < .001), more frequent ileal disease (33% vs 8.3%, *P* = .012), and a higher proportion of inflammatory behavior (93% vs 50%, *P* = .012). There were no significant differences in age, gender, or baseline disease activity measures between subgroups.

### Guselkumab induction effectiveness

**Figure 1:**
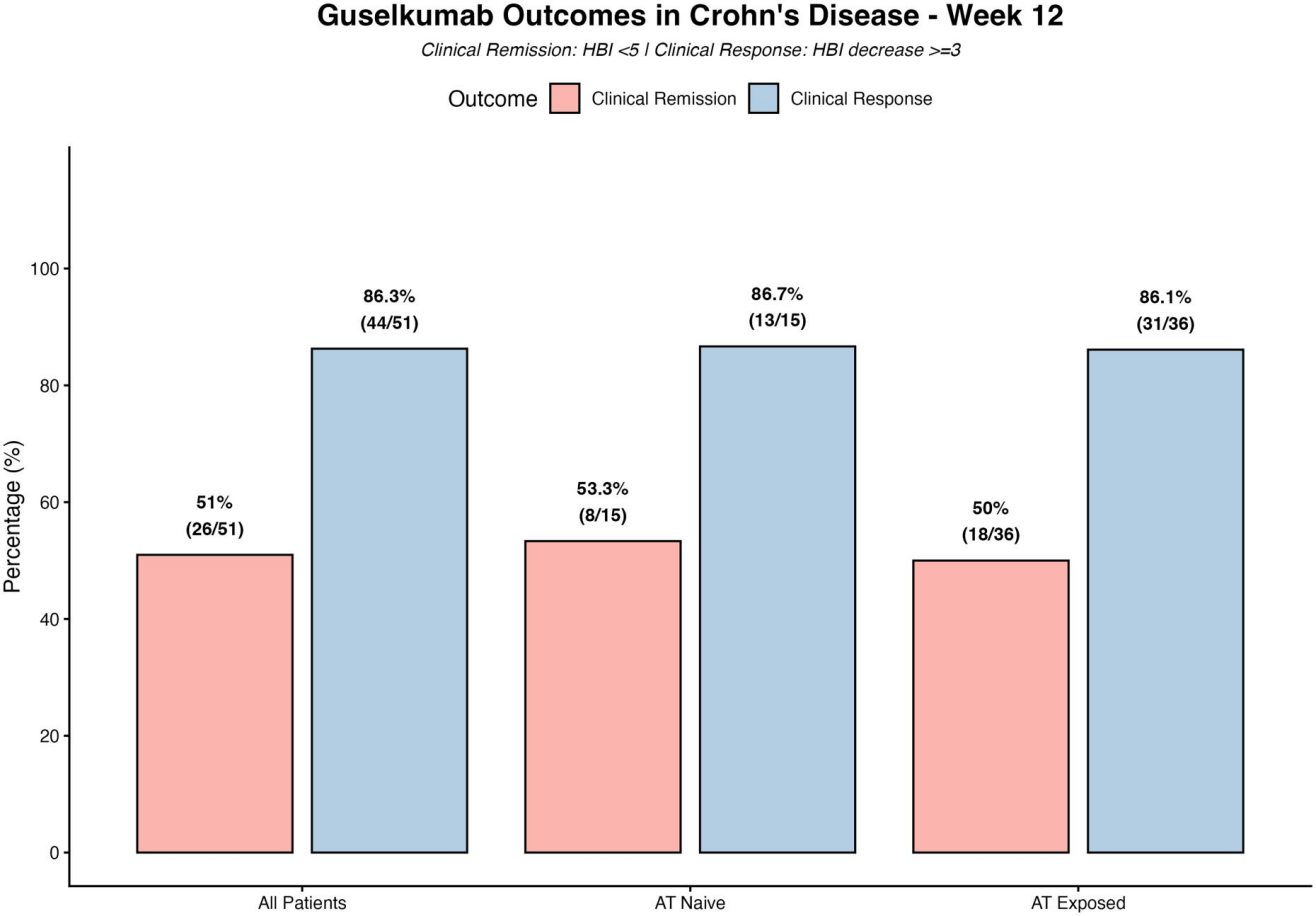
Guselkumab Week 12 clinical outcomes in Crohn’s disease. Bar chart showing rates of clinical remission (Harvey-Bradshaw Index <5) and clinical response (HBI decrease of 3 or more points from baseline) for all patients (*n* = 51), advanced therapy-naive (*n* = 15), and advanced therapy-exposed (*n* = 36) subgroups. Percentages with numerator/denominator are displayed above each bar.

Clinical remission (HBI <5) was achieved in 51% (26/51) and clinical response (HBI reduction of 3 or more) in 86% (44/51) at Week 12 (Table 2 and Figure 1). Effectiveness was consistent regardless of prior treatment history, with clinical remission rates of 53% (8/15) in AT-naive versus 50% (18/36) in AT-exposed patients (*P* > 0.99), and clinical response rates of 93% and 83%, respectively (*P* = .66). Biochemical remission (CRP <=5 mg/L and FCP <=250 μg/g) was achieved in 58% (26/45) of evaluable patients, with no significant difference between AT-naive and AT-exposed subgroups (58% vs 58%, *P* > 0.99). Significant reductions in inflammatory biomarkers were observed. Median CRP decreased from 8.5 mg/L (IQR 3.9-23.2) to 3.0 mg/L (IQR 1.4-11.0; *P* = .002) and median FCP decreased from 216 μg/g (IQR 70-801) to 80 μg/g (IQR 45-171; *P* < .001).

**Table 2:** Week 12 clinical outcomes for the Guselkumab CD cohort.

Outcome	Overall (*N* = 51)	AT-Naive (*N* = 15)	AT-Exposed (*N* = 36)	*P*-value
**Clinical remission (HBI <5)**	26 (51%)	8 (53%)	18 (50%)	>.99
**Clinical response (HBI drop >=3)**	44 (86%)	14 (93%)	30 (83%)	.66
**Biochemical remission**	26/45 (58%)	7/12 (58%)	19/33 (58%)	>.99

Among the 18 patients with prior ustekinumab exposure, clinical remission at Week 12 was achieved in 8 of 18 (44%), clinical response in 16 of 18 (89%), and biochemical remission in 9 of 18 (50%). Comparable outcomes were observed in the 33 ustekinumab-naive patients (clinical remission 18 of 33 [55%]; clinical response 28 of 33 [85%]). Prior ustekinumab exposure was not associated with reduced effectiveness on univariable analysis (Table S1). Among the 25 patients who did not achieve clinical remission at Week 12, 18 (72%) had elevated baseline CRP (>5 mg/L) and 15 (60%) had elevated baseline fecal calprotectin (>250 µg/g), suggesting that non-response was associated with persistent inflammation rather than predominantly functional symptoms. Across the cohort, baseline HBI correlated modestly with CRP (*r* = 0.38) and SES-CD (*r* = 0.37) but only weakly with fecal calprotectin (*r* = 0.07).

### Predictors of clinical remission

In univariable logistic regression, baseline HBI was a significant predictor of clinical remission (OR 0.77, 95% CI, 0.61-0.92, *P* = .011), with lower baseline HBI associated with higher remission rates. Baseline SES-CD was a significant predictor (OR 0.87, 95% CI, 0.74-0.99, *P* = .049), as was baseline CRP (OR 0.94, 95% CI, 0.89-0.98, *P* = .019) and number of prior advanced therapies (OR 0.71, 95% CI, 0.48-1.00, *P* = .063). No individual prior biologic class exposure significantly predicted remission (Table S1), though prior vedolizumab exposure showed a trend approaching significance (OR 7.89, 95% CI, 1.21-156, *P* = .066) that was not confirmed on multivariable analysis (OR 6.12, 95% CI, 0.45-167, *P* = .20). Notably, prior ustekinumab exposure, present in 35% of patients, did not negatively impact guselkumab outcomes. Disease behavior and AT-naive status did not significantly predict remission.

### Post-induction maintenance dose selection

The choice of subsequent maintenance regimen was made at the Week 12 induction assessment and reflects treating physician judgment of induction response. We therefore report this analysis here as an induction-window read-out rather than a maintenance outcome.

Following induction, 73% (37/51) commenced standard maintenance (100 mg every 8 weeks) while 27% (14/51) commenced intensified maintenance (200 mg every 4 weeks). Patients assigned to intensified maintenance had higher baseline disease severity (median HBI 8.5 vs 6.0, *P* = .008; median SES-CD 9.5 vs 6.0, *P* = .008), lower post-induction clinical remission rates (7.1% vs 68%, *P* < .001), lower post-induction clinical response rates (64% vs 95%, *P* = .013), and higher Week 12 fecal calprotectin (172 vs 64 μg/g, *P* = .004). In univariable analysis, higher baseline HBI (OR 1.37, 95% CI, 1.12-1.79, *P* = .006), higher baseline SES-CD (OR 1.27, 95% CI, 1.09-1.52, *P* = .004), failure to achieve post-induction remission (OR 0.04, 95% CI, 0.00-0.22, *P* = .003), and elevated Week 12 calprotectin (OR 1.01, 95% CI, 1.00-1.02, *P* = .010) were associated with selection of the intensified regimen.

### Safety

Guselkumab was well tolerated during the induction period. Adverse events were infrequent: one patient developed diverticulitis leading to discontinuation, one experienced eczema, one developed herpes zoster infection, and one had a relapse of peripheral spondyloarthropathy. No malignancies or other serious infections were reported during induction.

### Combination advanced therapy

Seven patients with multi-refractory ileo-colonic CD who had non-response or incomplete response post-induction received combination advanced therapy. The second agent added was infliximab in one patient and upadacitinib in six patients. Clinical response was achieved in the patient receiving the infliximab combination and in three of six patients receiving the upadacitinib combination.

### Propensity score–matched comparative effectiveness: Guselkumab versus risankizumab

#### Pre-matching cohort comparison

The combined cohort comprised 111 patients (51 guselkumab, 60 risankizumab), all with complete data for the 12 PSM covariates. Pre-matching, the cohorts were broadly comparable in age (GUS 32 [23-37] vs RIS 33 [26-44] years, *P* = .4), disease duration (85 [26-125] vs 62 [17-145] months, *P* = .6), baseline HBI (6.0 [5.0-8.0] vs 6.0 [4.0-7.0], *P* = .6), baseline CRP (8 [3-23] vs 8 [2-18] mg/L, *P* = .6), baseline FCP (239 [80-801] vs 350 [94-1250] μg/g, *P* = .3), and baseline SES-CD (6.0 [5.0-9.0] vs 8.0 [4.5-11.5], *P* = .3); (Table S2). A significant pre-matching difference was observed in baseline albumin (41 [39-44] vs 35 [31.5-40] g/L, *P* < .001). Baseline corticosteroid use was low in both groups (3 guselkumab, 4 risankizumab), with numbers too small for meaningful covariate adjustment.

#### Primary PSM analysis

Full matching retained all 111 patients (51 guselkumab, 60 risankizumab), with each patient assigned a weight reflecting the matched set structure. Post-matching covariate balance was assessed using standardized mean differences (Figure S1).

**Figure 2:**
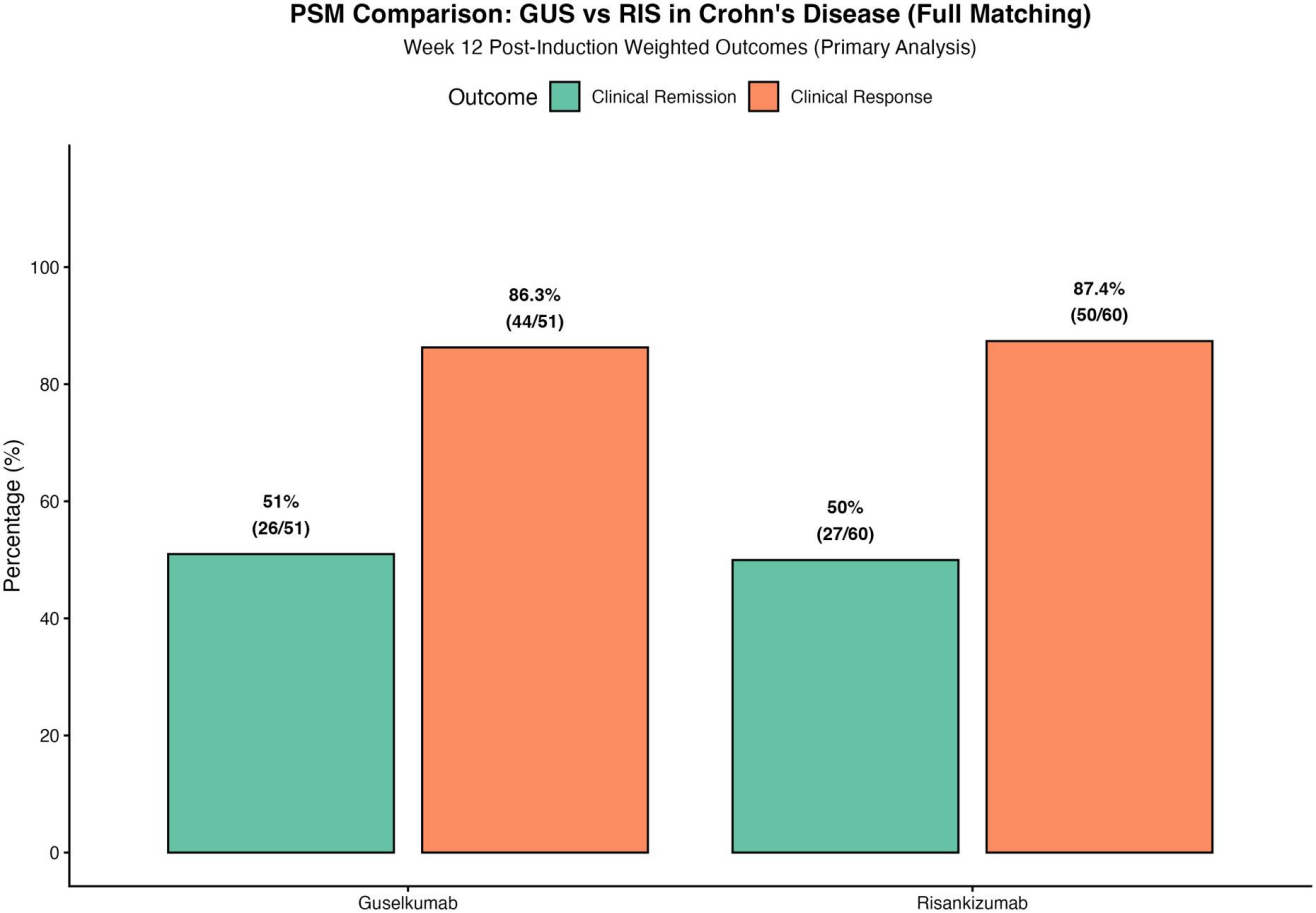
Propensity score-matched comparison of guselkumab versus risankizumab at Week 12 in Crohn’s disease. Bar chart showing weighted rates of clinical remission and clinical response following full (optimal) propensity score matching (51 guselkumab, 60 risankizumab). Matching controlled for 12 covariates including demographics, disease phenotype, baseline endoscopic and biochemical activity, surgical history, and current smoking status. No statistically significant differences were observed between agents for any outcome.

On weighted comparison following 12-covariate full optimal propensity score matching, guselkumab and risankizumab demonstrated comparable rates of clinical remission (51% [26/51] vs 50% [27/60], *P* > 0.9), clinical response (86.3% [44/51] vs 87.4% [50/60], *P* = .87), and biochemical remission (57.8% [26/51] vs 60.5% [32/60], *P* = .78), with no statistically significant differences (Table 3, Figure 2). Post-matching standardized mean differences for all 12 covariates were attenuated, including for current smoking status (pre-match SMD 0.45; post-match SMD 0.02; Figure S1). Both agents achieved significant within-group biomarker reductions: median CRP decreased from 7.6 (IQR 3-20) to 3 (IQR 1.2-11) mg/L with guselkumab (within-group *P* = .003; *n* = 47 paired) and from 5 (IQR 1-12) to 2.5 (IQR 1.8-5.3) mg/L with risankizumab (within-group *P* = .002; *n* = 56 paired); the between-group difference in CRP change was non-significant (weighted regression *P* = .79). Median fecal calprotectin decreased from 216 (IQR 65-837) to 80 (IQR 35-180) µg/g with guselkumab (within-group *P* = .0002; *n* = 35 paired) and from 334 (IQR 103-705) to 193 (IQR 73-293) µg/g with risankizumab (within-group *P* = .0002; *n* = 44 paired); the between-group difference was likewise non-significant (weighted regression *P* = .90).

**Table 3:** Propensity score–matched comparison of guselkumab and risankizumab: post-matching baseline, week 12 outcomes, and biomarker changes (primary analysis).

Characteristic	Guselkumab (*N* = 51)	Risankizumab (*N* = 60)	*P*-value
***Post-matching baseline (weighted)***			
**Age (years)**	32 (23, 37)	26 (22, 35)	—
**Male, *n* (wt%)**	33 (65)	36 (59)	—
**Disease duration (months)**	85 (26, 125)	77 (28, 122)	—
**Montreal L3, *n* (wt%)**	38 (75)	40 (72)	—
**Montreal B1, *n* (wt%)**	32 (63)	34 (57)	—
**Perianal disease, *n* (wt%)**	16 (31)	19 (29)	—
**Prior CD surgery, *n* (wt%)**	13 (25)	13 (34)	—
**Baseline HBI**	6 (5, 8)	6 (4, 7)	—
**Baseline CRP (mg/L)**	8 (3, 22.6)	6 (1, 15)	—
**Baseline Albumin (g/L)**	41 (39, 44)	37 (32, 40)	—
**Baseline FCP (**µ**g/g)**	239 (80, 801)	334 (30, 705)	—
**Baseline SES-CD**	6 (5, 9)	8 (4, 12)	—
**AT-naive, *n* (wt%)**	15 (29)	23 (29)	—
***Week 12 clinical outcomes (weighted)***			
**Clinical remission, *n* (wt%)**	26 (51)	27 (50)	>0.9
**Clinical response, *n* (wt%)**	44 (86.3)	50 (87.4)	0.866
**Biochemical remission, *n* (wt%)**	26 (57.8)	32 (60.5)	0.775
***Week 12 biomarker changes (paired)*** ^a^			
**CRP: baseline (mg/L)**	8.5 (3.7, 23.8)	5.7 (1, 14)	—
**CRP: week 12 (mg/L)**	3 (1.2, 11)	2.5 (1.3, 9.9)	—
**CRP: change (mg/L)**	−3.8 (−12.8, 0)	−1 (−9.6, 0.6)	>0.9
**CRP: within-group p**	0.002	0.002	—
**FCP: baseline (µg/g)**	216 (70, 801)	404 (92, 705)	—
**FCP: week 12 (µg/g)**	80 (45, 171)	118 (48, 193)	—
**FCP: change (µg/g)**	−139 (−735, 0)	−132 (−562, 0)	>0.9
**FCP: within-group *P***	<0.001	<0.001	—

Continuous variables: weighted median (IQR). Post-matching baseline dashes (—) indicate balance confirmed by Love plot (SMD). Outcome *P* value: weighted logistic regression. Between-group biomarker *P*: weighted linear regression.

a Biomarker analysis restricted to patients with paired baseline and week 12 data. Within-group *P*: Wilcoxon signed-rank test. wt% = weighted percentage.

#### Sensitivity analysis

The sensitivity analysis excluded 7 guselkumab patients with prior risankizumab exposure, yielding 104 patients (44 guselkumab, 60 risankizumab) for full matching. Post-matching covariate balance is shown in Figure S2. Results were consistent with the primary analysis, with no statistically significant differences between agents: clinical remission 54.5% (24/44) versus 58.2% (27/60) (*P* = .71); clinical response 88.6% (39/44) versus 92.5% (50/60) (*P* = .51); biochemical remission 65% (26/44) versus 76.8% (32/60) (*P* = .2).

## Discussion

This multicenter prospective cohort study provides real-world comparative effectiveness data for guselkumab versus risankizumab in Crohn’s disease, addressing a critical evidence gap in the absence of head-to-head randomized trials. Our findings demonstrate robust induction effectiveness of guselkumab in a refractory CD population and comparable short-term clinical outcomes between guselkumab and risankizumab in propensity score–matched analysis.

The effectiveness rates observed in our guselkumab cohort (clinical remission 51%; clinical response 86%) are broadly consistent with induction data from the pivotal GALAXI trials.^8^ Our cohort included substantial proportions of patients with stricturing (24%) or penetrating (14%) behavior and perianal disease (31%); features characteristic of the aggressive IBD phenotype described in the Middle East that typically predict poorer treatment outcomes.^19^^,^^20^ Guselkumab maintained robust efficacy in this difficult-to-treat population, with comparable outcomes between AT-naive and AT-exposed patients, mirroring findings from our Middle Eastern risankizumab cohort.^21^ Both agents significantly reduced CRP and fecal calprotectin from baseline, providing objective evidence of anti-inflammatory effect beyond clinical symptom improvement. Biochemical remission was achieved in 58% of evaluable guselkumab patients, consistent with the emerging literature demonstrating strong biomarker responses to selective IL-23 inhibitors.^8^^,^^25^

The comparative analysis against risankizumab represents the most novel aspect of this work. We employed full (optimal) propensity score matching with 12 covariates, including baseline endoscopic activity, biochemical markers (CRP and fecal calprotectin), prior CD surgery, and current smoking status, to ensure robust adjustment for disease severity.^18^ Full matching retains all patients in both arms, maximizing statistical power in a setting with modest sample sizes, and assigns weights to create balance across matched sets.^26^ This approach is methodologically well-established and preferable to 1:1 nearest-neighbor matching when sample sizes are limited, as it avoids discarding unmatched patients.^18^ Using this approach, our PSM analysis found comparable rates of clinical remission (51% vs 50%), clinical response (86% vs 87%), and biochemical remission (58% vs 60%) between guselkumab and risankizumab, with no statistically significant differences. Both agents achieved significant within-group reductions in CRP and fecal calprotectin, though between-group comparisons for both markers were non-significant, indicating comparable biochemical efficacy. A sensitivity analysis excluding prior risankizumab-exposed patients yielded consistent findings, confirming results were not confounded by within-class switching. Importantly, the comparable remission rates observed should not be interpreted as evidence of equivalence, as this study was not powered to detect clinically meaningful differences between treatments.

While both agents target the p19 subunit, they possess distinct molecular profiles: guselkumab’s native IgG1 Fc region allows for CD64 binding and potential direct engagement with IL-23-producing myeloid cells, whereas risankizumab’s LALA mutations minimize Fc-mediated effector functions.^9^^,^^27^ Whether these molecular differences translate into clinically meaningful outcome differences remains speculative and would require confirmation in adequately powered comparative studies. Our findings are concordant with published network meta-analyses, which currently provide the only indirect comparative evidence on selective interleukin-23 inhibitors in the absence of head-to-head trials. Recent network meta-analyses^11^^,^^12^^,^^28^ consistently rank guselkumab and risankizumab among the most effective induction agents for moderate-to-severe Crohn’s disease, without statistically significant separation between the two agents in any analysis that included both. Our propensity score–matched analysis provides the first direct real-world comparison between these agents in CD; the observed similarity in clinical remission (51% vs 50%), clinical response (86% vs 87%), and biochemical remission (58% vs 60%) is concordant with these indirect signals. Network meta-analyses are subject to recognized methodological limitations in the absence of head-to-head trial data, including transitivity assumptions across heterogeneous trial populations and designs. The convergence between direct real-world and indirect network meta-analytic evidence strengthens confidence that within-class differentiation between guselkumab and risankizumab at induction, if present, is of small magnitude. Notably, guselkumab effectiveness was preserved in patients with prior ustekinumab exposure. This observation is consistent with the proposed mechanistic advantage of selective IL-23p19 blockade over upstream IL-12/23 inhibition and supports clinical experience that prior IL-12/23 exposure does not preclude meaningful response to a selective IL-23p19 inhibitor.

Univariable regression identified baseline HBI, SES-CD, and CRP as significant predictors of clinical remission, with lower baseline disease activity associated with higher remission rates. The number of prior advanced therapies showed a trend approaching significance. No individual prior biologic class exposure significantly predicted remission; notably, prior ustekinumab exposure, present in over one-third of patients, did not attenuate guselkumab effectiveness. Prior vedolizumab exposure showed a non-significant trend on univariable analysis that was not confirmed on multivariable modeling. The analysis of maintenance dosing revealed that patients requiring dose intensification had significantly higher baseline disease severity and lower post-induction remission rates, supporting an objective, response-guided approach to maintenance dose selection. Higher baseline HBI was associated with reduced likelihood of clinical remission, but elevated objective markers of inflammation in the majority of non-remitters support an inflammatory rather than functional basis for treatment failure. The weak HBI-fecal calprotectin correlation reinforces the value of multi-modal disease assessment.

The unique characteristics of our population provide a rigorous stress test for novel therapies and begin to address the historical underrepresentation of Middle Eastern populations in pivotal IBD trials.^29^^,^^30^ Our methodology mirrors established real-world IBD comparative effectiveness studies with the addition of comprehensive PSM covariates addressing baseline disease severity. Several limitations warrant acknowledgment. First, this study was not powered to detect clinically meaningful differences between agents and no a priori sample size calculation was performed; the comparative analysis is therefore best interpreted as descriptive and hypothesis-generating. Although the observed between-group difference in clinical remission was small (51% vs 50% on weighted analysis), the 95% confidence interval around the absolute difference is wide (approximately −13 to +25 percentage points), and the cohort cannot exclude clinically meaningful between-arm differences in either direction. Comparable observed outcomes should not be interpreted as evidence of equivalence. Second, propensity score matching reduces bias from measured confounders but cannot account for unmeasured confounding. Despite comprehensive matching on 12 covariates, residual imbalances persisted in baseline albumin (although still largely within the normal range), reflecting some minor inherent differences between the two cohorts. Third, the primary clinical outcome (HBI) is a symptom-based index that correlates imperfectly with objective inflammation, particularly in patients with prior biologic exposure; we have therefore given emphasis to biomarker outcomes as complementary measures of treatment effect. Endoscopic outcome data were not available for the comparative analysis, though at 12 weeks, endoscopic changes may not yet reflect the full therapeutic effect. Fourth, follow-up is limited to the induction period; long-term maintenance data, including endoscopic outcomes, will be essential to determine whether pharmacokinetic differences impact durability of response or immunogenicity profiles. Fifth, concomitant oral corticosteroid use at baseline was minimal and was permitted at the clinician discretion in keeping with real-world practice; numbers were insufficient for inclusion as a PSM covariate or for meaningful subgroup analysis. Sixth, the observational study design is inherently susceptible to selection bias and confounding notwithstanding our matching approach, though the prospective data collection and real-world clinical setting may offer greater generalizability than controlled trials with restrictive eligibility criteria. The Harvey-Bradshaw Index does not directly capture perianal disease activity; perianal-specific outcomes were not the focus of this induction effectiveness analysis and should be evaluated in dedicated studies.

## Conclusion

Guselkumab demonstrated robust induction effectiveness in a complex Middle Eastern Crohn’s disease cohort, with propensity score–matched analysis revealing comparable remission rates to risankizumab, with no statistically significant differences. Treatment selection between these agents may be guided by practical considerations including route of administration, dosing flexibility, comparative cost, and regional availability. Larger multicenter comparative studies with endoscopic endpoints and longer follow-up are warranted to determine whether molecular differences between selective IL-23 inhibitors translate into meaningful clinical differentiation. Long-term outcome data, including durability of remission, are needed.

## Supplementary Material

otag061_Supplementary_Data

## Data Availability

The data that support the findings of this study are available from the corresponding author upon reasonable request. The data are not publicly available due to their sensitive nature, containing information that could compromise patient confidentiality and privacy.

## References

[1] Sewell GW , Kaser A. Interleukin-23 in the pathogenesis of inflammatory bowel disease and as a therapeutic target. J Crohns Colitis. 2022;16:ii3-19. 10.1093/ecco-jcc/jjab182 35553667 PMC9097674

[2] Bourgonje AR , Ungaro RC, Mehandru S, Colombel JF. Targeting the interleukin 23 pathway in inflammatory bowel disease. Gastroenterology. 2025;168:29-52.e3. 10.1053/j.gastro.2024.05.036 38945499

[3] Jairath V , Felquer MLA, Cho RJ. IL-23 inhibition for chronic inflammatory disease. The Lancet. 2024;404:1679-1692. 10.1016/S0140-6736(24)01750-1 39461795

[4] Duerr RH , Taylor KD, Brant SR, et al A genome-wide association study identifies IL23R as an inflammatory bowel disease gene. Science (1979). 2006;314:1461-1463. 10.1126/science.1135245 PMC4410764 17068223

[5] Verstockt B , Salas A, Sands BE, et al Alimentiv Translational Research Consortium (ATRC). IL-12 and IL-23 pathway inhibition in inflammatory bowel disease. Nat Rev Gastroenterol Hepatol. 2023;20:433-446. 10.1038/s41575-023-00768-1 37069321 PMC10958371

[6] Moschen AR , Tilg H, Raine T. IL-12, IL-23 and IL-17 in IBD: immunobiology and therapeutic targeting. Nat Rev Gastroenterol Hepatol. 2019;16:185-196. 10.1038/s41575-018-0084-8 30478416

[7] Peyrin-Biroulet L , Chapman JC, Colombel J-F, et al SEQUENCE Study Group. Risankizumab versus ustekinumab for moderate-to-severe Crohn’s disease. N Engl J Med. 2024;391:213-223. 10.1056/NEJMoa2314585 39018531

[8] Sandborn WJ , D’Haens GR, Reinisch W. Guselkumab for the treatment of Crohn’s disease: induction results from the phase 2 GALAXI-1 study and phase 3 GALAXI-2 and GALAXI-3 studies. Gastroenterology. 2024;167:1158-1174. 10.1053/j.gastro.2024.07.035 35134323

[9] Sands BE , Peyrin-Biroulet L, Kierkus J. Guselkumab binding to CD64+ IL-23–producing myeloid cells enhances potency for neutralizing IL-23 signaling. J Crohns Colitis. 2024;18:i465. 10.1093/ecco-jcc/jjad212.0165

[10] Ferrante M , Panaccione R, Baert F, et al Risankizumab as maintenance therapy for moderately to severely active Crohn’s disease: results from the multicentre, randomised, double-blind, placebo-controlled, withdrawal phase 3 FORTIFY maintenance trial. Lancet Lond Engl. 2022;399:2031-2046. 10.1016/S0140-6736(22)00466-4 35644155

[11] Shehab M , Alrashed F, Alrashidi A, et al Network meta-analysis: comparative efficacy of biologics and small molecules in the induction and maintenance of remission in Crohn’s disease. Aliment Pharmacol Ther. 2025;62:472-482. 10.1111/apt.70295 40717455

[12] Barberio B , Gracie DJ, Black CJ, Ford AC. Efficacy of biological therapies and small molecules in induction and maintenance of remission in luminal Crohn’s disease: systematic review and network meta-analysis. Gut. 2023;72:264-274. 10.1136/gutjnl-2022-328052 35907636

[13] Sands BE , Irving PM, Hoops T, et al Ustekinumab versus adalimumab for induction and maintenance therapy in biologic-naive patients with moderately to severely active Crohn’s disease: a multicentre, randomised, double-blind, parallel-group, phase 3b trial. Lancet Lond Engl. 2022;Jun 11; 399:2200-2211. 10.1016/S0140-6736(22)00688-2 35691323

[14] Sands BE , Peyrin-Biroulet L, Loftus EV, et al VARSITY Study Group. Vedolizumab versus adalimumab for moderate-to-severe ulcerative colitis. N Engl J Med. 2019;381:1215-1226. 10.1056/NEJMoa1905725 31553834

[15] Ferrante M , D’Haens G, Jairath V, et al Efficacy and safety of mirikizumab in patients with moderately-to-severely active Crohn’s disease: a phase 3, multicentre, randomised, double-blind, placebo-controlled and active-controlled, treat-through study. The Lancet. 2024;404:2423-2436. 10.1016/S0140-6736(24)01762-8 39581202

[16] Gordon M. Network meta-analyses in IBD: pitfalls and promise for clinicians. Therap Adv Gastroenterol. 2026;19:7562848251408758. 10.1177/17562848251408758 PMC12804628 41551496

[17] Lenti MV , Dolby V, Clark T, et al A propensity score-matched, real-world comparison of ustekinumab vs vedolizumab as second-line treatment for Crohn’s disease: the cross pennine study II. Aliment Pharmacol Ther. 2022;55:856-866. 10.1111/apt.16742 34935160 PMC9305775

[18] Austin PC. An introduction to propensity score methods for reducing the effects of confounding in observational studies. Multivar Behav Res. 2011;46:399-424. 10.1080/00273171.2011.568786 PMC3144483 21818162

[19] Ahmed HA , Alzaabi MN, Swaid TK, et al Evolving clinical burden of inflammatory bowel disease in the United Arab Emirates: a two-decade analysis of diagnoses and disease severity. Frontline Gastroenterol. 2026;17:312-321. 10.1136/flgastro-2025-103345

[20] Al Atrash E , Mohammad SM, Mohammad S, Al Ghafli H, Miqdady M, Quraishi MN. Accelerating burden of paediatric IBD in UAE: phenotypes and treatment patterns at a single Centre (2012-2024). Frontline Gastroenterol. 2026;17:322-330. 10.1136/flgastro-2025-103397

[21] Alzaabi MN , Swaid TK, Ahmed HA, et al Real-World effectiveness of risankizumab in Crohn’s disease: outcome data for an IL-23 inhibitor from the Middle east. Crohns Colitis. 2025;360:otaf062. 10.1093/crocol/otaf062 PMC12674788 41346973

[22] von Elm E , Altman DG, Egger M, Pocock SJ, Gøtzsche PC, Vandenbroucke JP; STROBE Initiative Strengthening the reporting of observational studies in epidemiology (STROBE) statement: guidelines for reporting observational studies. BMJ. 2007;335:806-808. 10.1136/bmj.39335.541782.AD. 17947786 PMC2034723

[23] R Core Team. R: A Language and Environment for Statistical Computing [Internet]. Vienna, Austria: R Foundation for Statistical Computing; 2025. Available from: https://www.R-project.org/

[24] Ho D , Imai K, King G, Stuart EA. MatchIt: nonparametric preprocessing for parametric causal inference. J Stat Softw. 2011;42:1-28. 10.18637/jss.v042.i08

[25] Rubin DT , Allegretti JR, Panés J, et al Guselkumab in patients with moderately to severely active ulcerative colitis (QUASAR): phase 3 double-blind, randomised, placebo-controlled induction and maintenance studies. The Lancet. 2025;405:33-49. 10.1016/S0140-6736(24)01927-5. 39706209

[26] Stuart EA , Green KM. Using full matching to estimate causal effects in nonexperimental studies: examining the relationship between adolescent marijuana use and adult outcomes. Dev Psychol. 2008;395-406. 10.1037/0012-1649.44.2.395 18331131 PMC5784842

[27] Feagan BG , Sandborn WJ, D’Haens G. The role of selective IL-23 blockade in inflammatory bowel disease: mechanistic insights and therapeutic implications. J Crohns Colitis. 2023;17:1897-1908. 10.1093/ecco-jcc/jjad13237738465

[28] Singh S , Murad MH, Yuan Y, et al Comparative efficacy of advanced therapies for management of moderate-to-severe Crohn’s disease: 2025 AGA evidence synthesis. Gastroenterology. 2025;169:1516-1536. 10.1053/j.gastro.2025.08.032 41114682 PMC12614769

[29] Pathiyil MM , Jena A, Venkataramana Raju AK, Omprakash TA, Sharma V, Sebastian S. Representation and reporting of diverse groups in randomised controlled trials of pharmacological agents in inflammatory bowel disease: a systematic review. Lancet Gastroenterol Hepatol. 2023;8:1143-1151. 10.1016/S2468-1253(23)00193-0 37832569

[30] Shehab M , Al-Hindawi A, Goodwin SW, et al Closing the Middle east and North Africa representation gap in clinical trials in inflammatory bowel disease. Lancet Gastroenterol Hepatol. 2026;11:89-91. 10.1016/S2468-1253(25)00346-2 41314228

