# Supplementary material for: Real-world effectiveness of guselkumab for induction of remission in Crohn’s disease: a prospective multicenter cohort study with propensity score–matched comparison to risankizumab
Source: Crohns Colitis 360. 2026 Jun 19;8(2):otag061. doi: 10.1093/crocol/otag061 (PMC13316425; doi:10.1093/crocol/otag061)

**Supplementary Material**

**Real-World Effectiveness of Guselkumab for Induction of Remission in Crohn’s Disease: A Prospective Multicentre Cohort Study with Propensity Score-Matched Comparison to Risankizumab**

**Contents**

Supplementary Table 1. Univariable predictors of clinical remission at Week 12 in Crohn's disease

Supplementary Table 2. Pre-Matching Baseline Comparison

Supplementary Figure 1. Covariate balance before and after propensity score matching for the 12-covariate model (including current smoking status): Primary analysis (all guselkumab-treated Crohn's disease patients)

Supplementary Figure 2. Covariate balance before and after propensity score matching for the 12-covariate model (including current smoking status): Sensitivity analysis (excluding guselkumab patients with prior risankizumab exposure)

**Supplementary Table 1.** Univariable Predictors of Clinical Remission (Guselkumab CD Cohort)

| **Characteristic** | **OR** | **95% CI** | **p-value** |
| --- | --- | --- | --- |
| **Age (years)** | 0.96 | 0.90, 1.01 | 0.13 |
| **Gender** |  |  |  |
| **F** | — | — |  |
| **M** | 1.06 | 0.33, 3.39 | 0.92 |
| **Disease Duration (months)** | 1.00 | 0.99, 1.01 | 0.47 |
| **Baseline HBI** | 0.77 | 0.61, 0.92 | 0.011 |
| **Baseline SES-CD** | 0.87 | 0.74, 0.99 | 0.049 |
| **Baseline CRP (mg/L)** | 0.94 | 0.89, 0.98 | 0.019 |
| **Baseline Faecal Calprotectin (ug/g)** | 1.00 | 1.00, 1.00 | 0.19 |
| **Number of Prior ATs** | 0.71 | 0.48, 1.00 | 0.063 |
| **Perianal Disease** |  |  |  |
| **No** | — | — |  |
| **Yes** | 0.65 | 0.19, 2.15 | 0.49 |
| **Prior CD Surgery** |  |  |  |
| **Yes** | — | — |  |
| **No** | 0.86 | 0.24, 3.05 | 0.81 |
| **AT Naive** |  |  |  |
| **Yes** | — | — |  |
| **No** | 0.88 | 0.26, 2.94 | 0.83 |
| **Prior Anti-TNF Exposure** |  |  |  |
| **Yes** | — | — |  |
| **No** | 1.52 | 0.50, 4.79 | 0.46 |
| **Prior Ustekinumab Exposure** |  |  |  |
| **Yes** | — | — |  |
| **No** | 1.50 | 0.47, 4.87 | 0.49 |
| **Prior Vedolizumab Exposure** |  |  |  |
| **Yes** | — | — |  |
| **No** | 7.89 | 1.21, 156 | 0.066 |
| **Prior Upadacitinib Exposure** |  |  |  |
| **Yes** | — | — |  |
| **No** | 2.29 | 0.40, 17.7 | 0.37 |

*Abbreviations: CI = Confidence Interval, OR = Odds Ratio, AT = Advanced Therapy, HBI = Harvey-Bradshaw Index, SES-CD = Simple Endoscopic Score for Crohn’s Disease, CRP = C-reactive protein. Median (Q1, Q3); n (%).*

**Supplementary Table 2.** Pre-Matching Baseline Comparison

| **Characteristic** | **GUS (N=51)** | **RIS (N=60)** | **p-value** |
| --- | --- | --- | --- |
| **Age (years)** | 32 (23, 37) | 33 (26, 44) | 0.4 |
| **Disease Duration (months)** | 85 (26, 125) | 62 (17, 141) | 0.6 |
| **Baseline HBI** | 6.0 (5.0, 8.0) | 6.0 (4.0, 7.0) | 0.6 |
| **Baseline CRP (mg/L)** | 8 (3, 23) | 8 (2, 18) | 0.6 |
| **Baseline Albumin (g/L)** | 41.0 (39.0, 44.0) | 35.0 (31.8, 40.0) | <0.001 |
| **Baseline FCP (ug/g)** | 239 (80, 801) | 350 (94, 1,198) | 0.3 |
| **Baseline SES-CD** | 6.0 (5.0, 9.0) | 8.0 (4.8, 11.3) | 0.3 |
| **Number of Prior ATs** | 2.00 (1.00, 4.00) | 2.00 (1.00, 3.50) | 0.5 |
| **Gender: Female** | 18 (35%) | 24 (40%) | 0.6 |
| **Montreal L3 (Ileocolonic)** | 38 (75%) | 40 (67%) | 0.2 |
| **Montreal B1 (Inflammatory)** | 32 (63%) | 34 (57%) | 0.4 |
| **Perianal Disease** | 16 (31%) | 19 (32%) | >0.9 |
| **AT Naive** | 15 (29%) | 23 (38%) | 0.3 |
| **Prior CD Surgery** | 13 (25%) | 13 (22%) | 0.6 |
| **Prior Anti-TNF Exposure** | 30 (59%) | 31 (52%) | 0.5 |
| **Prior Ustekinumab Exposure** | 18 (35%) | 24 (40%) | 0.6 |
| **Current Smoker** | 5 (10%) | 14 (23%) | 0.078 |

*Continuous variables: median (IQR). Categorical: n (%). p-values: Wilcoxon rank-sum, Pearson Chi-squared, or Fisher exact test. AT = advanced therapy; GUS = guselkumab; RIS = risankizumab.*

**Supplementary Figure 1.** Covariate balance before and after propensity score matching: Primary analysis (all guselkumab-treated Crohn's disease patients)


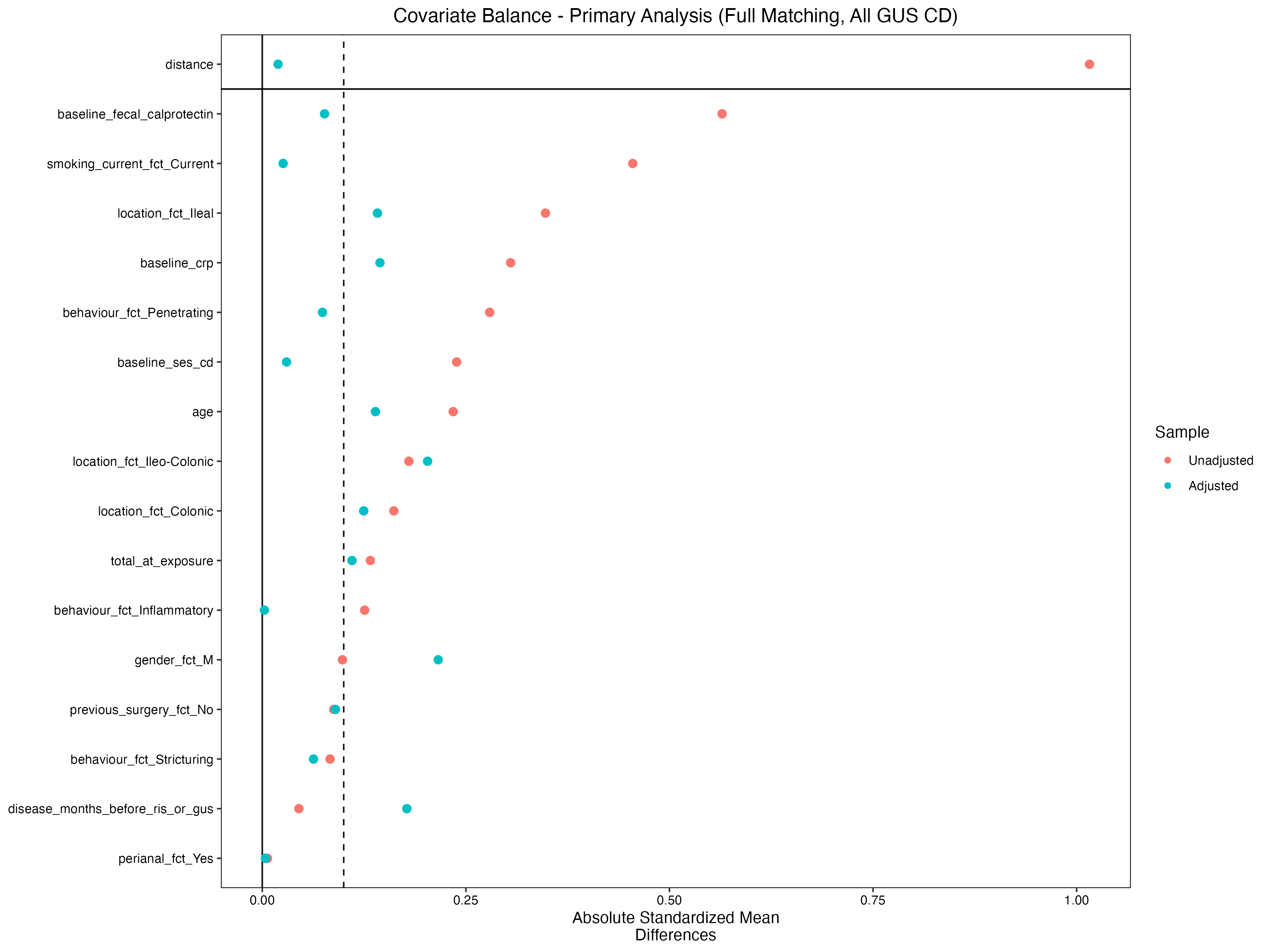


**Supplementary Figure 2.** Covariate balance before and after propensity score matching: Sensitivity analysis (excluding guselkumab patients with prior risankizumab exposure)


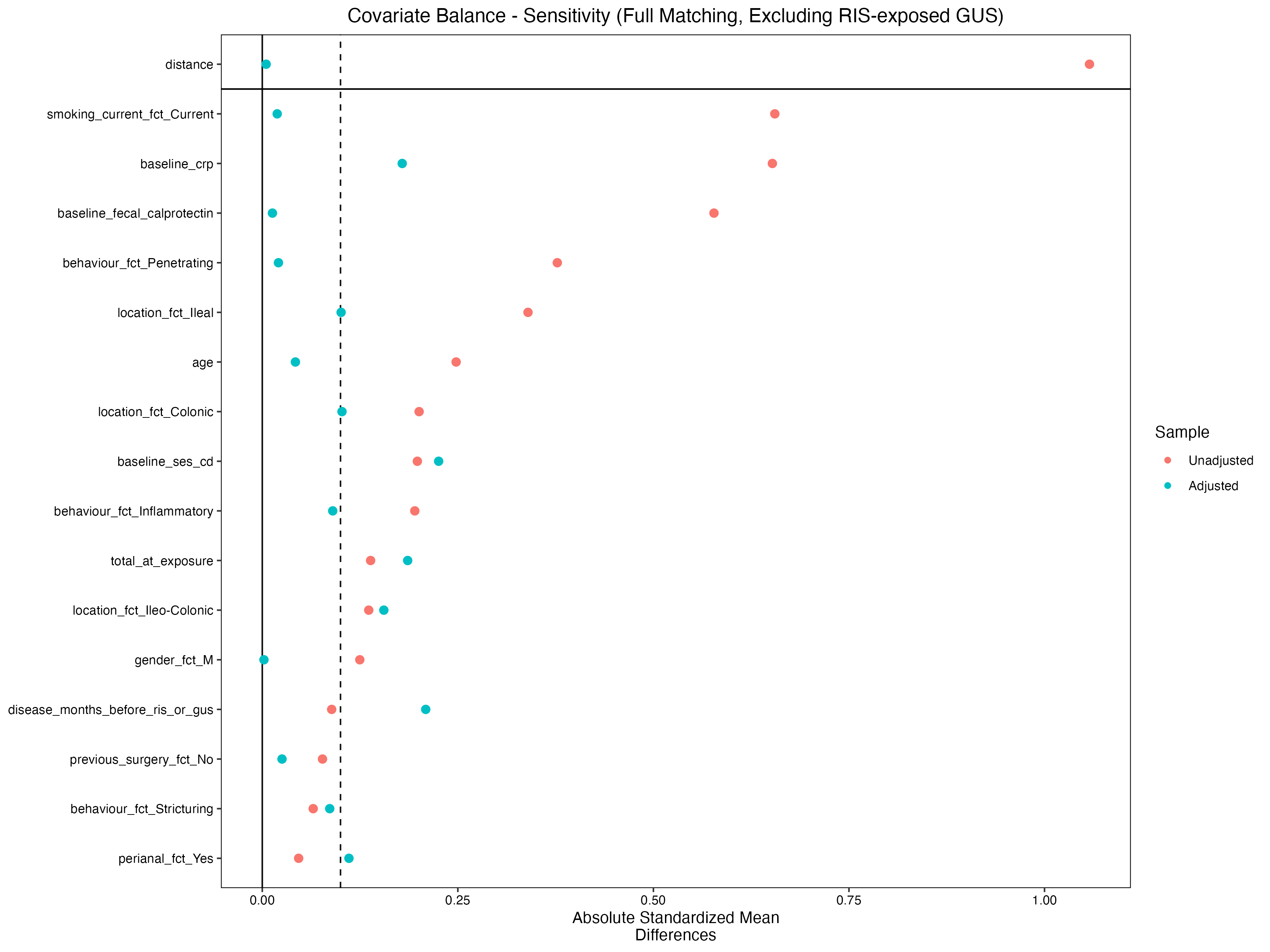

Supplement: otag061_Supplementary_Data [file otag061_supplementary_data.zip › Supplementary File GUS CD v2.docx]
